# Metric networks for enhanced perception of non-local semantic information

**DOI:** 10.3389/fnbot.2023.1234129

**Published:** 2023-08-09

**Authors:** Jia Li, Yu-qian Zhou, Qiu-yan Zhang

**Affiliations:** College of Applied Mathematics, Chengdu University of Information Technology, Chengdu, Sichuan, China

**Keywords:** multi-branch siamese network, non-local information perception, semantic information capture, cross-source, cross-view

## Abstract

**Introduction:**

Metric learning, as a fundamental research direction in the field of computer vision, has played a crucial role in image matching. Traditional metric learning methods aim at constructing two-branch siamese neural networks to address the challenge of image matching, but they often overlook to cross-source and cross-view scenarios.

**Methods:**

In this article, a multi-branch metric learning model is proposed to address these limitations. The main contributions of this work are as follows: Firstly, we design a multi-branch siamese network model that enhances measurement reliability through information compensation among data points. Secondly, we construct a non-local information perception and fusion model, which accurately distinguishes positive and negative samples by fusing information at different scales. Thirdly, we enhance the model by integrating semantic information and establish an information consistency mapping between multiple branches, thereby improving the robustness in cross-source and cross-view scenarios.

**Results:**

Experimental tests which demonstrate the effectiveness of the proposed method are carried out under various conditions, including homologous, heterogeneous, multi-view, and crossview scenarios. Compared to the state-of-the-art comparison algorithms, our proposed algorithm achieves an improvement of ~1, 2, 1, and 1% in terms of similarity measurement Recall@10, respectively, under these four conditions.

**Discussion:**

In addition, our work provides an idea for improving the crossscene application ability of UAV positioning and navigation algorithm.

## 1. Introduction

In recent years, with the development of deep learning (LeCun et al., 2015), traditional computer vision tasks witnessed significant progress (Liu Z. et al., 2023; Shi et al., 2023b). Among them, metric learning, which focuses on matching and retrieval (Kaya and Hasan, 2019) has achieved remarkable advancements in accuracy and reliability. Scholars' attention has gradually shifted from the traditional homologous (the images come from the same sensor) visual matching task to the multi-source (the images come from two or more sensors) image matching task (Yang et al., 2022), and even the multi-modal (datas come from different types, such as images and text) matching task (Hu et al., 2022; Xu et al., 2023).

Traditional image matching methods, such as SIFT and SURF, have been widely used to extract stable key points and local descriptors from images, which are then compared to compute matching similarity (Ma et al., 2021). Later, feature point extraction methods combined with deep learning emerged. For example, SuperPoint (Landrieu and Boussaha, 2019) utilizes convolutional neural networks to efficiently extract feature points and descriptors in an end-to-end trainable manner. DenseVLAD (Torii et al., 2015) is a feature extraction and matching method based on Bag of visual words model. It extracts local features in images by dense sampling and calculates their similarity through vector quantization method. Although these methods have shown promising results in homology matching tasks, it is more and more difficult to extract common features because of the increasing differences among the data, and their reliability diminishes significantly when confronted with the complexities of multi-source, complex, or multi-modal data. Up to now, there is still no literature that can solve these challenges at the same time.

Metric learning aims to learn a function that quantifies the similarity between diverse data sources, perspectives, or modalities. These sources can originate from different sensors, devices or fields, such as visible light images, infrared images, radar images, etc. Multiple views (Hassani and Khasahmadi, 2020) can arise from different viewpoints or shooting locations, such as images from varying cameras, videos, lidar point cloud data, and so on. Furthermore, multimodality encompasses different modalities, including text, images, audio, video, and others. The objective of metric learning is to acquire a distance metric function that minimizes the distance between samples belonging to the same class while maximizing the distance between samples from different classes in a low-dimensional embedding space. The primary challenge in metric learning lies in effectively modeling the similarity relationships across different sources, perspectives, or modalities and appropriately fusing the information from diverse data sources, perspectives, or modalities.

Metric learning serves as the foundation of image matching, demonstrating remarkable capability in learning image similarities and greatly advancing various tasks (Ma et al., 2021; Wang D. et al., 2022). For example, in the task of face recognition (Boutros et al., 2022), deep learning methods effectively capture the facial information under complex conditions, enabling accurate identification of individuals based on semantic attributes. Similarly, in vehicle re-identification (Shen et al., 2023), the metric learning framework facilitates reliable screening of complex multi-view positive samples, leading to precise consensus decision-making despite variations in multi-sensor data. A prominent network structure that implements the metric learning framework is the siamese neural network, exemplified by MatchNet (Han et al., 2015). Comprising two identical neural networks sharing weights and parameters, the siamese neural network processes different input data with the goal of calculating similarity or dissimilarity between input pairs.

Siamese neural network has been widely used in face recognition, target tracking, semantic matching, recommendation system and other fields, yielding favorable outcomes. Particularly, for remote sensing image matching task, the same scene often contains multiple types of remote sensing data captured simultaneously, such as repeated data from the same source, satellite data, UAV aerial data, and even ground street view data (Zhai et al., 2017). Usually, the corresponding siamese neural network structure is established for each category. However, this approach can introduce interference from a specific branch during model training, limiting the adaptability of the model. In this regard, the establishment of a multi-branch siamese neural network to learn the relationship among multi-source data plays a crucial role in promoting the ability of image perception from a single data source. Moreover, during the learning process of learning multi-branch data, the similarity measure of the model can provide supplementary information through the third branch to supplement and eliminate the impact of the difference of data sources.

In the structure of Siamese Neural Network, Convolutional Neural Network (CNN) is one of the most critical parts. However, conventional CNNs rely on local perceptions for feature extraction, which suffer from limitations such as excessive emphasis on local regions and limited interaction between different regions. In contrast, non-local feature perception, unlike traditional CNNs, considers the correlation among all locations within the feature map when computing features for each location (Tu et al., 2020). This approach utilizes a global similarity measure to evaluate the relevance of an input feature to all other locations and assigns computed weights to the corresponding feature representation within the feature map. Consequently, it captures more semantic information across the global context, enhancing the expressive power of the features. Non-local feature perception enables the capture of global and long-range correlation information, thereby improving the expressiveness of the features. It finds application in various image processing tasks, including image classification, object detection, and semantic segmentation. However, non-local operations are computationally demanding, consuming additional computing resources and time. Furthermore, they are sensitive to noise or outliers in the input feature map and thus require special handling. Semantic Enhancement (Hao et al., 2020) in deep learning refers to a learning-based technique that enhances a model's capacity to perceive and extract semantic information from input data, thereby improving its performance and robustness. In deep learning, enhancing semantic information typically involves increasing the depth and width of the model to improve its expressiveness and ability to perceive and extract semantic information. Alternatively, methods such as Attention Mechanism (Guo et al., 2022) and Gate Mechanism (Khanh et al., 2020) can be employed to prioritize important semantic information in the input data, thus improving the accuracy and robustness of the model. Additionally, techniques such as Non-local Networks consider the relationships between different locations within the input data to enhance the comprehension of semantic information by the model.

In this study, we seek to answer the question: How can the limitations of current image matching methods be addressed through the use of a multi-branch siamese neural network model? We propose a multi-branch siamese neural network model to address the challenges of metric learning in complex tasks, with a specific focus on remote sensing images, UAV aerial images, and ground street-view images. To overcome the limitations of traditional neural networks in perceiving global features, we introduce the incorporation of multi-level long-distance features to enhance the information perception capabilities of the siamese network branches. Furthermore, we tackle the challenge of matching difficulty arising from significant differences in data sources within complex environments. To address this, we propose a semantic information enhancement and measurement model that leverages the characteristics of multi-source image semantic information to establish a metric discrimination model. The main contributions of this paper can be summarized as follows:

(1) We construct a multi-branch siamese neural network model. By employing the multi-branch siamese network, we can understand the physical properties of salient objects in different network branches. The features extracted from these branches are embedded in the same feature space, establishing an attribute consistency relationship between different branches. Through information interaction during the learning process of the multi-branch model, we enhance the network's ability to handle heterogeneous remote sensing effects.
(2) We introduce a feature-aware model for capturing non-local information. Features at different levels are extracted from various levels of the network. By fusing these features with the output features of the backbone network, we obtain the relationship between different non-local locations in the data. This allows us to utilize the information that positively impacts the similarity measurement in the feature extraction network more effectively.
(3) Based on semantic enhancement, we achieve semantic alignment in the multi-branch siamese networks. Moreover, we utilize the common target in the matching data as a bridge to connect multiple sources and views. Therefore, we can extract deeper semantic attributes from images and enhance the alignment ability of information attributes between branches. As a result, semantic information can be combined more effectively.

In Section 2, the related research progress is reviewed. In Section 3, we introduce our proposed method. In Section 4, we provide experimental verification of our method, outlining the setup, data used, and results achieved. Section 5 concludes the paper, summarizing our findings and suggesting areas for future work.

## 2. Related works

### 2.1. Metric learning

Metric Learning is a fundamental branch of machine learning that plays a crucial role in various computer vision tasks, including image retrieval (Yan et al., 2021), face recognition (Li M. et al., 2022), person re-identification (Gu et al., 2022a), etc.

The nearest neighbor algorithm, as a classic metric learning method, determines the class of a sample based on the distances between samples. In classification tasks, the algorithm identifies the closest training samples to a test sample and assigns the test sample to the corresponding class. Siamese networks are highly effective metric learning techniques used for comparing pairs of input samples. Comprising two identical neural networks with shared parameters, siamese networks generate a similarity score through a distance metric function. They have proven successful in tasks such as image matching and face recognition (Gu et al., 2022b; Li M. et al., 2022). Distance metric learning is a key aspect of metric learning, aiming to learn a function that can measure the distance between samples. Various methods exist for distance metric learning, including prototype-based methods (Gu et al., 2022b), metric matrix-based methods (Price et al., 2022), and maximum margin-based methods (Li X. et al., 2022). Among these, Max-Margin Metric Learning (MMML) has emerged as a classic technique maximizing the distances between different classes while minimizing the distances within the same class.

While existing methods focus on homologous data, this paper addresses the challenges of metric learning in multi-source data scenarios. Therefore, it investigates the measurement problem in multi-source complex scenes, aiming to explore mechanisms for enhancing metric learning in such scenarios.

### 2.2. Remote sensing image retrieval

Remote sensing image retrieval methods (Zhou et al., 2018) can be broadly categorized into: content-based retrieval and context-based retrieval. Content-based retrieval relies on essential image characteristics, such as color, texture, and shape, while context-based retrieval considers the relationships between images, such as location, size, orientation, etc. Each method possesses distinct advantages and disadvantages, and the appropriate approach should be selected based on the specific requirements of the application.

In the field of remote sensing image retrieval, research methods can be classified into traditional methods (Deselaers et al., 2008) and deep learning methods (Saritha et al., 2019). Traditional methods encompass feature extraction techniques, including color histogram, texture features, SIFT, SURF, as well as classic machine learning approaches like Bag of Words and TF-IDF. Although these methods have achieved some success in previous research, their effectiveness in addressing the complexity and diversity of remote sensing images remains limited. Consequently, deep learning methods have gained significant popularity in recent years for remote sensing image retrieval. Among deep learning methods, Convolutional Neural Networks (CNNs) have emerged as a powerful tool, exhibiting remarkable success in image processing and finding increased adoption in remote sensing image retrieval (Liu et al., 2018; Liu X. et al., 2023). CNNs have been leveraged for feature extraction, image classification, and image retrieval, resulting in notable improvements in retrieval accuracy and efficiency. For instance, Li et al. (2021) proposed a deep retrieval network based on a multi-branch architecture, demonstrating superio performance in large-scale remote sensing image retrieval tasks. This network consists of two parallel branches: a global feature branch, a local feature branch, and a similarity fusion module. Experimental results demonstrate that the proposed method outperforms state-of-the-art remote sensing image retrieval methods in terms of accuracy and speed. Liu et al. (2020) introduced a multi-scale deep feature learning method based on the siamese network for remote sensing image retrieval. This approach incorporates multi-scale feature learning and a multi-task loss function to enhance retrieval accuracy and efficiency. Experimental results highlight its excellent retrieval performance on various remote sensing datasets. Furthermore, Huang et al. (2023) proposed a remote sensing image retrieval method based on deep multi-scale fusion. Their approach employs a novel multi-scale fusion strategy to capitalize on the complementarity between global and local features, thereby improving the accuracy and robustness in remote sensing image retrieval. Experimental results demonstrate that the proposed method surpasses other state-of-the-art approaches on different datasets.

While the aforementioned methods primarily address the issue of homologous images, more complex scenarios involving multi-source, multi-view, and multi-modal conditions, present different challenges. Incorporating these factors into the image dissimilarity framework poses a greater challenge in establishing meaningful metric mappings.

### 2.3. Multi-source image matching

With the emergence of deep learning, an increasing number of researchers have started applying it to multi-source image matching. Prominent deep learning methods in this context include Convolutional Neural Networks (CNNs), Recurrent Neural Networks (RNNs), and Generative Adversarial Networks (GANs), among others. Multi-source image matching approaches based on deep learning typically utilize deep neural networks to extract feature representations from images and employ them for image matching. These methods offer a key advantage in learning superior feature representations through the end-to-end training mechanism of deep neural networks, leading to higher matching accuracy.

For instance, Xu et al. (2019) proposed a cross-modal retrieval method based on deep adversarial metric learning. Their approach employs two GAN models: one for generating supplementary modal features and the other for incorporating adversarial losses in the embedding space. Additionally, a sample difficulty mining mechanism (Schroff et al., 2015) is employed to enhance the robustness and generalization capability of the training samples. Experimental results demonstrate that the proposed method outperforms other approaches in cross-modal retrieval tasks. In summary, this paper's primary contribution lies in introducing adversarial losses and sample difficulty mining mechanisms to enhance the robustness and generalization ability of cross-modal retrieval methods. When compared to traditional metric learning-based methods, this approach achieves superior performance on various modal datasets. Additionally, the GAN models in this study presents a novel solution to challenges such as data augmentation and feature fusion in other cross-modal applications. Similarly, Hu et al. (2020) proposed an unsupervised knowledge distillation method for learning from unlabeled data in cross-modal hashing. Their approach adopts an adversarial learning framework between an encoder network and a decoder network. The encoder network maps cross-modal data into a shared latent space, while the decoder network reconstructs data from this latent space. This method undergoes evaluation on multiple cross-modal datasets and demonstrates its superior performance when compared to existing methods.

In complex scenes, the extraction of consistent semantic information from images plays a crucial role in enhancing the model's ability to discriminate features.

## 3. Methods

Image matching plays a crucial role in remote sensing image processing, encompassing various types of images captured by diverse sensors, perspectives, times, spectral ranges, and resolutions. The objective of image matching is to identify the same object across multiple sources. Solving this problem is of paramount importance for applications such as 3D reconstruction, change detection, resource management, and environmental monitoring of the Earth's surface.

In this paper, we propose a multi-branch network model to address the challenge of multi-scene matching in remote sensing images. Our approach focuses on designing a discriminative network that establishes consistent matching relationships considering various data sources and conditions. By leveraging the capabilities of the multi-branch network, we aim to improve the accuracy and reliability of multi-scene matching in remote sensing images.

### 3.1. Multi-branch siamese neural networks

Metric learning has seen remarkable advancements, particularly with the widespread utilization of two-branch siamese neural networks (Chicco, 2021) for feature extraction and similarity evaluation in image pairs. These networks aim to determine matching outcomes based on the relationship between feature representations in sample pairs (Wang and Liu, 2021).

Traditional two-branch metric learning networks utilize a contrastive loss function to learn the consistency relationship between samples and positive samples, as well as distinct features for negative samples to enhance discriminative capability. However, the learning process of positive and negative relationships in the contrastive loss function can exhibit uncertainty, posing challenges in network learning. In order to solve this problem, a ternary loss function is proposed. By considering both positive and negative samples to guide the learning of the network, it can converge to the expected direction.

Furthermore, when faced with data samples exhibiting significant intra-class variations and limited inter-class differences, the task becomes increasingly challenging. In such cases, the two-branch siamese neural network commonly employs an anchor-based approach (Schroff et al., 2015) to address this issue. This strategy involves clustering samples into groups and selecting key samples as anchors to simplify the metric learning process, a widely adopted method in practice. Different branches of the network serve distinct roles in the metric matching task, such as multi-source image metric or positive and negative sample branch learning. In the context of similarity measurement for multi-source remote sensing images, a typical scenario involves matching satellite images, UAV images, and ground view images.

**Figure 1:**
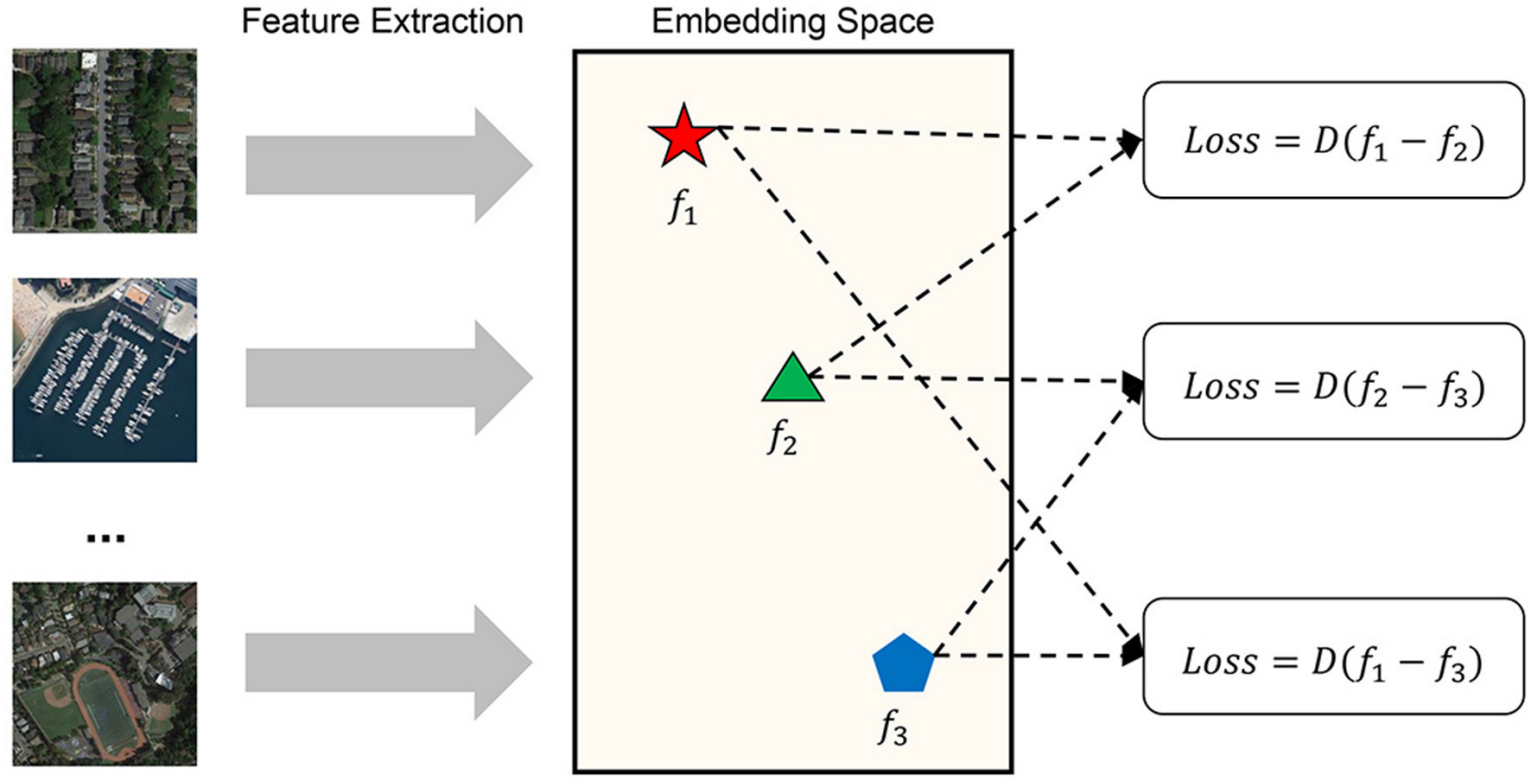
Multi-branch siamese neural network (the framework of image similarity measurement consists of two main parts: feature extraction and similarity calculation. It uses the backbone network of feature extraction to capture the depth feature descriptor of the image. In feature extraction, an abstract feature space is formed. In this space, the features of all samples are distributed according to certain rule, the positive samples are clustered into clusters, and negative samples and positive samples form a certain distance. The similarity measurement model determines the abstract distance of the samples in the space, judges whether the sample pairs meet the similarity threshold, and finally gives the judgment result of the image similarity measurement).

In this study, we propose a novel siamese neural network model designed to accurately determine the similarity of satellite-UAV images. Our approach involves constructing a multi-branch siamese neural network that employs ResNet (He et al., 2016) as the backbone network to extract visual features from remote sensing images, aerial images, and ground view images. To effectively distinguish positive samples, we utilize the contrast loss function across different branches of the network. The proposed multi-branch siamese neural network is shown in Figure 1. The contrastive loss function is expressed as follows:


$$\begin{array}{l} D\left(f_{1},f_{2}\right)=\frac{1}{2N}\overset{N}{\underset{n=1}{\sum }}yd^{2}+\left(1-y\right)max{\left(margin-d,0\right)}^{2}, \end{array}$$

where *f*_1_ and *f*_2_ represent two samples to be measured, *d* represents the 2-norm between samples, *y* = 1 indicates a sample match, *y* = 0 indicates a sample mismatch, *N* is the number of samples and *margin* is the set threshold.

Establishing an end-to-end all-input multi-branch siamese network for merging tasks holds great significance as it allows for the integration of information from different branches. During training, every two branches take turns to participate in training. This enables the model to measure similarities between any two branches, regardless of the data inputs involved.

### 3.2. Non-local information sensing

Extensive research has focused on feature extraction methods for remote sensing images (Zhang et al., 2022). In the context of multi-source image matching, effectively capturing features that can be used to measure similarity poses a significant challenge. This challenge is particularly evident in various scenarios encountered in multi-source matching tasks:

(a) **General multi-source imagery:** Scenes exhibit significant differences, which can be effectively addressed using traditional siamese neural networks.
(b) **Aviation-remote sensing image matching:** Scenes differ while retaining similar detailed textures.
(c) **View difference image matching:** Objects remain consistent, while details and textures vary noticeably.

To address these challenges, we propose a novel strategy of non-local feature selection and mining. We introduce a multi-level information capture module into the feature extraction backbone network to retain intermediate features. By establishing non-local models for perceiving texture, object, and semantic information, our approach enables multi-scene and multi-scale measurement by leveraging full-scale non-local features.

**Figure 2:**
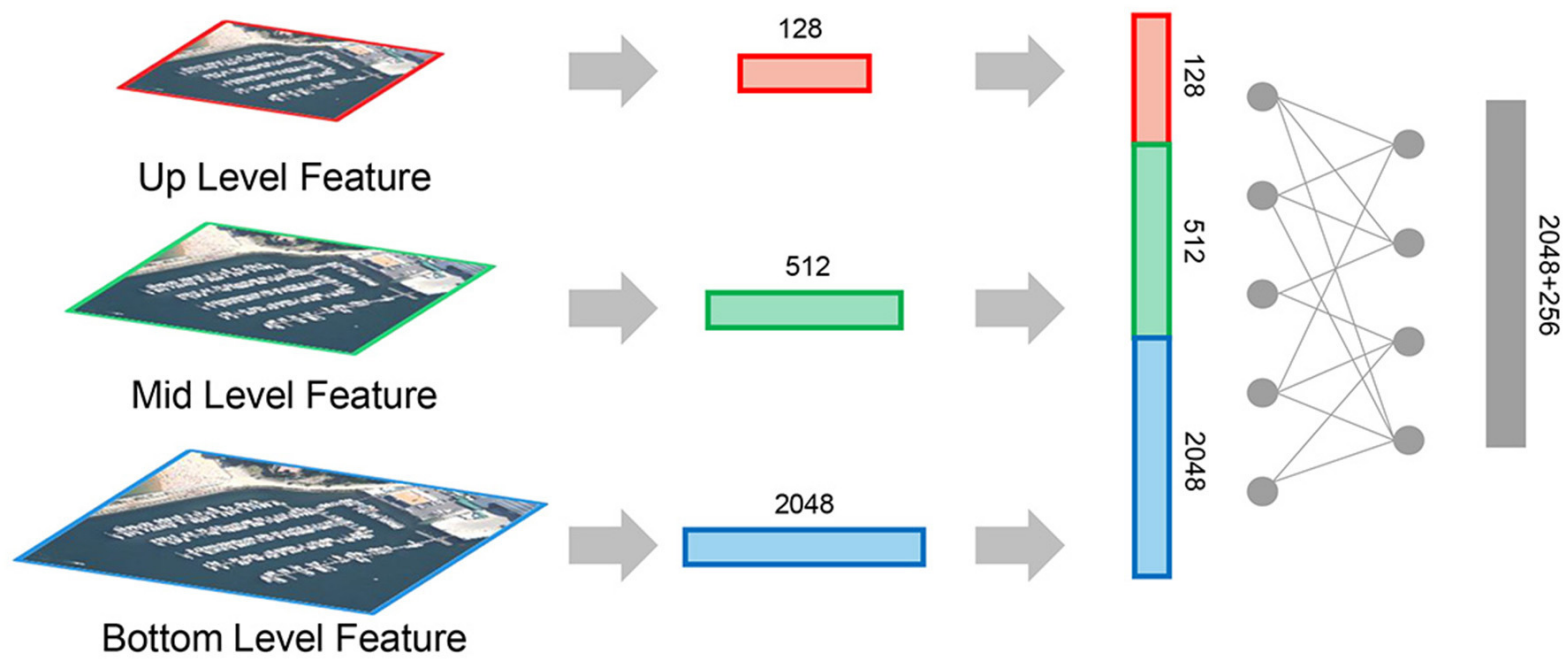
Non-local feature selection and mining structure diagram (abstract features of different levels are fused into a 256-dimensional feature vector, while the output feature dimension of the backbone network is 2,048. The non-local strategy adopted in this paper is to fuse the two parts of features by concat, and then reduce them by full connection, and finally obtain the features with the same dimension as the original output feature).

The structure of non-local feature selection and mining is illustrated in Figure 2. The information is captured from the bottom-up layer of the backbone network, and the resulting multilevel non-local features are mapped into a unified space by using a fully connected mapping layer. Through multi-scale non-local feature fusion, we obtain more robust visual features. In the metric space, the descriptive ability of different scale features improves, enabling the final deep features to express detailed distribution information while containing rich objects and their corresponding semantic descriptions. In addition, the interaction of non-local information within the feature extraction network positively influences the perception of local information. Through fusion, the process effectively compensates for the limitations of single-scale features, enhancing the overall descriptive power of the network.

### 3.3. Semantic information enhancement

Deep convolutional networks serve as a common tool for feature extraction, wherein feature vectors are mapped into the semantic space through fully connected layers or other techniques to enhance the model's understanding and representation of semantic information. Augmenting semantic information has the potential to improve a model's capacity to perceive and extract semantic information from input data, resulting in enhanced performance and robustness. This augmentation has been successfully applied in various image processing and natural language processing tasks, including image classification, object detection, semantic segmentation, machine translation, and question answering systems. However, the increased complexity and computational requirements associated with the model often demand additional computational resources and time.

In highly complex scenarios characterized by significant viewpoint or data source differences, conventional siamese network models demonstrate its inherent limitations in metric learning (Zheng et al., 2020a).

**Figure 3:**
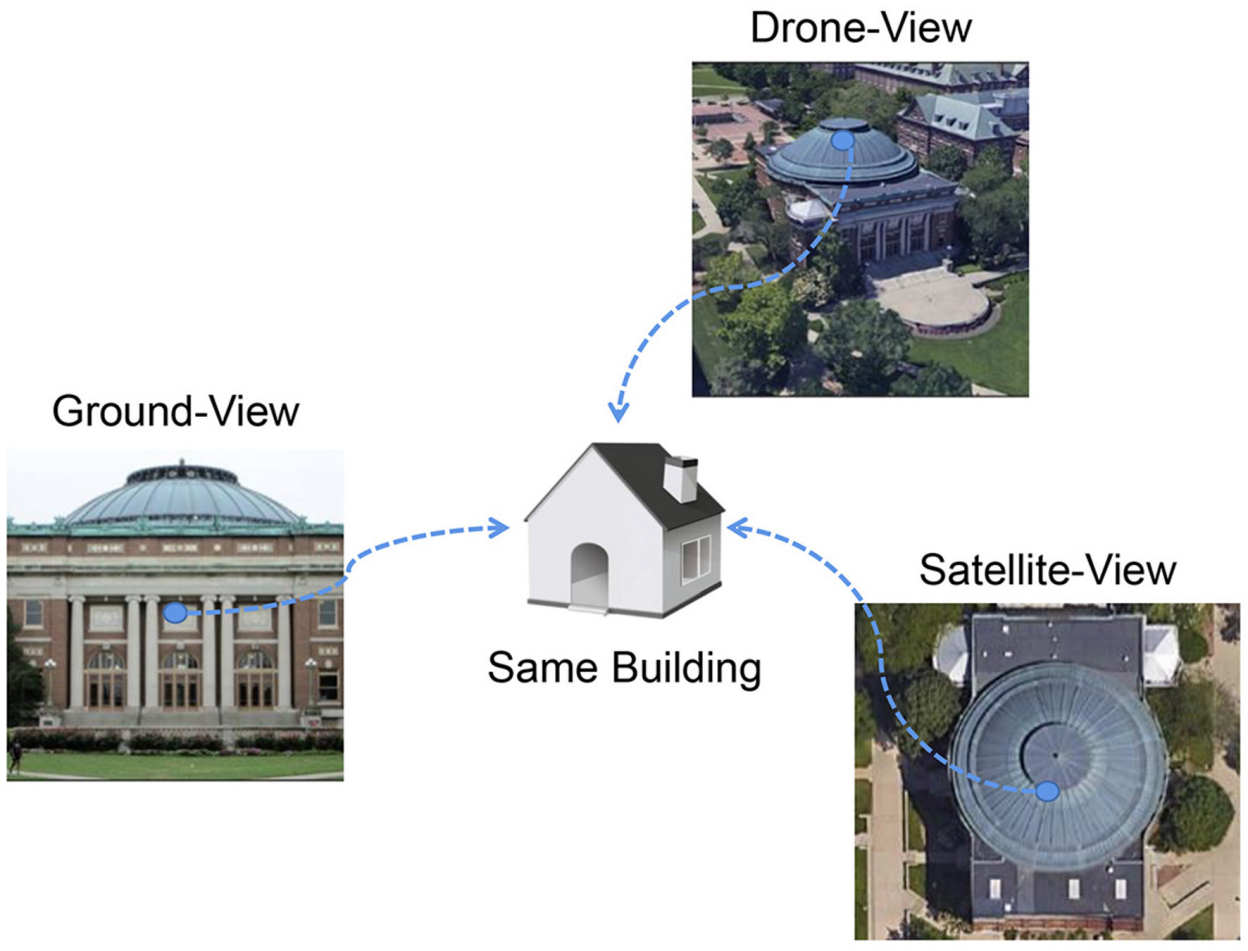
Semantic information capture.

To overcome this challenge, we introduce the concept of utilizing a common target within the matching data as a bridge to connect multiple sources and views (Figure 3). Within the structure of the multi-branch siamese neural network, we design a model for semantic information perception and enhancement. This model simplifies the intricate multi-source and multi-view task by focusing on discriminating salient objects based on captured semantic information.

The semantic information capture and enhancement model are implemented within different branches of the siamese neural network. Specifically, we employ an attention enhancement model, which can be represented as follows:


$$\begin{array}{l} M_{s}=\sigma \left(f^{7\times 7}\left(\left[AvgPool\left(F\right);MaxPool\left(F\right)\right]\right)\right), \end{array}$$

Where *F* represents the features entering the attention module, *f*(·) represents convolution, AvgPool and Max Pool represent mean pooling and max pooling, respectively. Let σ denote the activation function.

In this study, attention enhancement is applied to different feature dimensions, enabling the acquisition of salient object representations. Subsequently, similarity matching is enhanced by using the attention consistency between different branches.

## 4. Experiment

In order to verify the proposed algorithm, experimental tests are carried out under the conditions of homologous, heterogeneous, multi-view and cross-view, respectively, in this chapter. These four conditions are from the easier to the more advanced, especially the last one which is very challenging. Its purpose is to verify the effects of different modules and loss functions on metric learning based on the same baseline in different scenarios. Finally, it can be verified that the accuracy of matching can be effectively improved by adding the modules proposed in this manuscript. In all experiments, MatchNet, with RESNET50 as the backbone network, is used as the baseline. All experiments were completed on NVIDIA RTX3090 with batchsize of 14 and optimized by SGD. We mainly use recall@10 (the higher the better) to evaluate the algorithm, and other indicators can also be used as a reference (Everingham et al., 2010).

### 4.1. Homologous image matching

Homologous means that the training set and the prediction set come from the same sensor, and the image matching will not be affected by the sensor. Remote sensing image retrieval is one of the representative tasks. With the rapid development of remote sensing earth observation technology, the amount of remote sensing image data received and archived has increased exponentially. However, the limited breakthroughs in the content understanding and fast retrieval technology for remote sensing images severely restrict their utilization and efficiency. Enhancing the efficiency of homologous matching tasks in remote sensing image classification and archiving remains a significant challenge. In this section, we conduct experiments on the publicly available AID dataset (Xia et al., 2017) to evaluate the performance of the proposed algorithm on the homologous matching problem. AID is a new large-scale aerial image dataset, by collecting sample images from Google Earth imagery. The dataset has a number of 10,000 images within 30 classes.

Table 1 shows the performance of different algorithms in homologous image matching. The experimental results clearly demonstrate that the proposed algorithm surpasses the siamese neural network and outperforms the comparison algorithms in terms of accuracy. By incorporating non-local feature fusion, our algorithm surpasses the attention mechanism method by capturing more semantic information across the global scope and enhancing the expressive power of features. Furthermore, compared to VGG-MatchNet, the augmentation of semantic information has a more pronounced enhancement effect compared to non-local information, resulting in an average accuracy improvement of ~10%.

**Table 1:** Results of homologous image matching.

**Method**	**Recall@10**	**Recall@5**
Vgg-MatchNet	71.63%	36.54%
ResNet-MatchNet	77.84%	33.85%
SENet (Hu J. et al., 2018)	78.59%	35.43%
CBAM (Woo et al., 2018)	88.95%	38.79%
SOSNet (Tian et al., 2020)	89.62%	39.01%
SOLAR (Ng et al., 2020)	90.28%	38.52%
Proposed method	**91.04%**	**41.25%**

Bold values indicate the best results.

### 4.2. Multi-source image matching

The training set and prediction set of multi-source matching come from different sensors, but the perspective is the same. The difference between data sets is mainly reflected in the errors of illumination and color brought about by sensors. In its early stages, multi-source image matching found applications in aircraft flight guidance. By retrieving the satellite database consistent with the aerial image during flight, corresponding geographic position information could be obtained, enabling the mapping of aircraft perspective images to the geographic map. In this section, based on the newly released hospital image matching data set LA500 (Liu et al., 2022) in recent years, the proposed method is verified. LA500 is a simulated dataset based on the Google Earth Software. In this simulated dataset, typical city views of bare ground in the outskirts, serried buildings, streets, and vehicles are included in it. It contains 500 aerial images.

Table 2 shows the performance of different algorithms in multi-source image matching. The experimental results reveal that as the problem shifts toward multi-source image matching, the accuracy of all models decreases due to the existence of domain gaps. However, our proposed algorithm still outperforms the attention mechanism method. The incorporation of semantic enhancement improves the model's ability to perceive and extract similar semantic information from different source data, resulting in enhanced performance and robustness. Moreover, the supplementation of non-local information ensures the reliability of semantic information extracted through semantic enhancement. These factors collectively contribute to the improved modeling capabilities of our algorithm for multi-source matching and enable it to mitigate the differences inherent in multi-source images to a certain extent.

**Table 2:** Results of multi-source image matching.

**Method**	**Recall@10**	**Recall@5**
Vgg-MatchNet	62.98%	36.85%
ResNet-MatchNet	60.31%	33.73%
SENet (Hu J. et al., 2018)	63.44%	35.69%
CBAM (Woo et al., 2018)	63.98%	38.65%
SOSNet (Tian et al., 2020)	63.84%	39.12%
SOLAR (Ng et al., 2020)	61.23%	38.09%
Proposed method	**65.32%**	**41.04%**

Bold values indicate the best results.

### 4.3. Multi-view and multi-source image matching

In multi- view and multi- source scenarios, the training set and the prediction set come from different sensors and have different perspectives, but the perspective span is small, for example, Satellite → UAV. Multi-source multi-view scene matching is a computer vision algorithm used for localization and navigation, leveraging image data from multiple sensors or viewpoints to determine the camera's position and orientation. The algorithm aims to match the input image with a pre-established map or reference image to determine the position of the camera in the world coordinate system.

This approach finds applications in various fields, including robot navigation, unmanned vehicles, augmented reality, etc. By incorporating information from multiple viewpoints or sensors, the accuracy and robustness of localization can be improved, leading to more reliable localization and navigation capabilities. Based on the University-1652 dataset (Zheng et al., 2020a), this paper carries out tests to verify the reliability of the proposed method in multi-source and multi-view problems. University-1652 is a multi-view multi-source benchmark for drone-based geolocalization, It contains data from three platforms, i.e., synthetic drones, satellites and ground cameras of 1,652 university buildings around the world. The experimental results are shown in Table 3.

**Table 3:** Results of multi-view and multi-source image matching.

**Method**	**Recall@1**	**Recall@5**	**Recall@10**	**Recall@top1%**	**mAP**
Contrastive	40.69%	61.43%	72.94%	71.33%	44.15%
Triplet	51.95%	71..56%	79.66%	59.43%	54.68%
LPN (Wang et al., 2021)	74.83%	**89.77%**	91.43%	91.98%	77.46%
Proposed method	**74.93%**	89.38%	**92.49%**	**92.84%**	**78.93%**

Bold values indicate the best results.

Experimental results show that the proposed algorithm achieves an accuracy of 78.93% (mAP) in the university1652 data set, outperforming the comparison algorithms. Multi-source and multi-view matching pose challenges due to image differences caused by external factors such as illumination and sensors from different sources, as well as feature differences resulting from different viewpoints. By using the partition measure, the multi-view image is relatively weaker in the central part, LPN obtains better multi-view reliability and achieves the accuracy second only to the proposed algorithm. Compared with LPN, the proposed method can fully exploit the multi-view invariance of partitions, therefore a better accuracy effect than LPN is achieved. The proposed method obtains the ability to cope with view changes in three ways. Firstly, by considering the relationship between different non-local positions in the input data through feature fusion, the proposed algorithm enhances its multi-view reliability. Secondly, it utilizes a common target in the matching data as a bridge connecting multiple sources and views. Thirdly, through semantic enhancement, the algorithm extracts deeper semantic attributes from images, enabling reliable image matching even in the presence of complex view changes.

### 4.4. Multi-source cross-view image matching

In the scenario of multi-source and cross-view, the training set and the prediction set come from different sensors and different perspectives, and the perspective span is large, for example, Satellite → Street View. Cross-view geo-localization (Zhai et al., 2017) is a challenging computer vision task that aims to estimate the exact geographical location of a view based on its features. By training a model using a dataset with known geographical information, and then mapping new views to geographical locations using this model, cross-view geo-localization finds applications in image-based localization and navigation systems. These systems rely on different viewpoints or images to determine the location of cameras or observers in the geographic space, enabling city navigation, map annotation, and augmented reality.

Challenges in Cross-view geo-localization include viewpoint differences, illumination changes, occlusions, scale variations, and dataset diversity. To overcome these challenges, researchers usually employ data augmentation techniques, multimodal information fusion, deep learning models, or domain adaptation methods across datasets. Cross-view geo-localization provides valuable insights into the relationship between image data and geospace. In this paper, CVUSA (Zhai et al., 2017) is used to test the effect of the proposed algorithm in the cross-view matching task. CVUSA (Workman et al., 2015) is A large dataset containing millions of pairs of ground-level and aerial/satellite images from across the United States. The experimental results are shown in Table 4.

**Table 4:** Results of multi-source cross-view image matching.

**Method**	**Recall@1**	**Recall@5**	**Recall@10**	**Recall@top1%**
CVM-Net (Hu S. et al., 2018)	18.80%	44.42%	57.47%	91.54%
Instance Loss (Zheng et al., 2020b)	43.91%	66.38%	74.58%	91.78%
LPN (Wang et al., 2021)	85.79%	95.38%	96.98%	99.41%
CVFT (Shi et al., 2020)	61.43%	84.69%	90.49%	99.02%
DWDR (Wang T. et al., 2022)	75.62%	90.45%	93.60%	98.60%
Proposed method	**86.94%**	**95.99%**	**97.43%**	**99.57%**

Bold values indicate the best results.

Experimental results show that the proposed methods outperform the baseline algorithm LPN, achieving approximately a 1% recall improvement on Recall@1-10. The cross-view matching task entails not only extreme view differences but also significant data distribution inconsistencies. In this regard, starting from understanding images from different data sources. Firstly, we leverage multi-branch networks to comprehend the physical attributes of salient objects in different siamese network branches. By employing local feature fusion and semantic enhancement in the branch backbone network, we effectively enhance the branch network's ability to understand these attributes. Subsequently, we embed the features extracted from different branches into the same feature space and establish attribute consistency relationships between different branches. Finally, in the learning process of multi-branch model, additional information is provided by other branches to eliminate the influence of data source differences and realize consistency measurement under cross-view conditions. Through these three steps, our model effectively addresses the associated challenges and achieves superior results in cross-view image matching.

## 5. Conclusion

In this research, we have presented a novel framework for metric learning in complex scenarios involving multi-source and multi-view data. Our proposed approach addresses the limitations of traditional metric learning methods by introducing a multi-branch siamese neural network model. This model utilizes positive and negative samples to guide the learning process, enabling effective handling of highly complex multi-view problems with information from intermediate branches. In addition, we have proposed a non-local information perception model, which adapts to the measurement decision-making for different scenarios. Furthermore, we have employed a semantic information perception and enhancement model to establish a robust mapping relationship between multi-source and multi-view models. This integration of semantic information enhances the reliability of measurement decisions and improves the overall performance of the proposed algorithm.

Moving forward, our future work will focus on applying the proposed algorithm in practical projects, specifically in the domain of UAV positioning and navigation. (Shi et al., 2023a; Tian et al., 2023; Wang et al., 2023). Given the computing power limitations of UAVs, we aim to optimize the model by reducing its complexity and memory occupation while maintaining real-time reasoning speed. By lightening the model and improving its practical application ability, we can enhance its effectiveness in real-world scenarios. Additionally, we will explore other related problems and continue to advance the field of multi-source and multi-view matching and positioning.

## Data availability statement

The original contributions presented in the study are included in the article/supplementary material, further inquiries can be directed to the corresponding author.

## Author contributions

All authors listed have made a substantial, direct, and intellectual contribution to the work and approved it for publication.
